# Seroprevalence of SARS‐CoV‐2 in Livestock in Sarawak, East Malaysia

**DOI:** 10.1155/tbed/9927316

**Published:** 2026-07-21

**Authors:** Cheng Siang Tan, Vaenessa Noni, Ahmad Syatir Tahar, Carolynna Buyau James, George Bobby, Jackie Peter

**Affiliations:** ^1^ Department of Paraclinical Sciences, Faculty of Medicine and Health Sciences, Universiti Malaysia Sarawak, Kota Samarahan, Sarawak, 94300, Malaysia, unimas.my; ^2^ Therapeutic Research Centre, Faculty of Medicine and Health Sciences, Universiti Malaysia Sarawak, Kota Samarahan, Sarawak, 94300, Malaysia, unimas.my; ^3^ Centre for Tropical and Emerging Diseases, Faculty of Medicine and Health Sciences, Universiti Malaysia Sarawak, Kota Samarahan, Sarawak, 94300, Malaysia, unimas.my; ^4^ Sarawak Veterinary Diagnostic Laboratory, Department of Veterinary Services Sarawak, Kota Samarahan, Sarawak, 94300, Malaysia

**Keywords:** livestock, SARS-CoV-2, seroprevalence, surrogate virus neutralization test

## Abstract

Livestock remain an understudied component of the severe acute respiratory syndrome coronavirus 2 (SARS‐CoV‐2) ecology in Southeast Asia despite close and frequent contact with humans in smallholder farming systems. This cross‐sectional study investigated the seroprevalence of SARS‐CoV‐2 neutralizing antibodies in livestock in Sarawak, Malaysian Borneo. Archived plasma samples collected between June and September 2024 from 180 livestock animals, including pigs, cattle, buffalo, sheep, and goats, were obtained from the Sarawak Veterinary Diagnostic Service Laboratory and tested using the GenScript cPASS surrogate virus neutralization test (sVNT), a species‐independent assay detecting antibodies that block the SARS‐CoV‐2 spike receptor‐binding domain (RBD)–ACE2 interaction. Overall, 19.4% (35/180) of samples were seropositive using the manufacturer‐recommended cut‐off. Seroprevalence varied significantly by species, with the highest prevalence observed in goats (48.6%), followed by sheep (28.6%), cattle (11.4%), buffalo (8.3%), and pigs (2.6%), and also differed by sampling location but not by age group or sex. Most positive samples exhibited low‐level inhibition values near the assay cut‐off, suggesting weak or transient neutralizing antibody responses. Multivariable logistic regression identified species as the primary factor independently associated with seropositivity. Collectively, these findings are consistent with sporadic spillback exposure of SARS‐CoV‐2 from humans into livestock in Sarawak, without evidence of widespread or sustained transmission among livestock populations. Periodic, risk‐based surveillance of livestock remains warranted to monitor potential changes in host susceptibility as SARS‐CoV‐2 continues to evolve.

## 1. Introduction

The severe acute respiratory syndrome coronavirus 2 (SARS‐CoV‐2), the causative agent of coronavirus disease 2019 (COVID‐19), emerged in late 2019 and rapidly escalated into a global pandemic with substantial public health and socioeconomic consequences [1]. The pandemic has been described as a major One Health crisis because a presumed bat‐origin virus crossed into humans and subsequently spilled back into multiple animal species with frequent human contact, including companion animals [2–9], wildlife [10, 11], and captive and farmed animals [12, 13]. Previously, the highly divergent B.1.641 Lineage of SARS‐CoV‐2 was detected in white‐tailed deer (*Odocoileus virginianus*) in Canada, with evidence of sustained evolution in deer and probable deer‐to‐human transmission. Such findings indicate that animals may be secondary reservoirs or mixing hosts for new variants under suitable ecological and epidemiological conditions, underscoring the need for integrated human–animal surveillance [14].

Compared with companion animals and wildlife, the roles of livestock in SARS‐CoV‐2 transmission remain less well described. Experimental infection studies suggest that livestock species are generally unlikely to act as major maintenance hosts, although susceptibility varies by species. A study integrating livestock of multispecies (cattle, sheep, alpacas, goats, rabbits, and horses) found limited evidence of infection after experimental challenge, with no viral shedding detected, reflecting the limited epidemiological significance of these species [15]. Pigs generally appear to have low permissiveness to SARS‐CoV‐2 infection, while findings for small ruminants are more variable, including reports of natural serological exposure in sheep and goats [16]. To date, sustained livestock‐to‐livestock transmission has not been clearly established. However, livestock remain underrepresented in SARS‐CoV‐2 maintenance, despite being commonly managed in husbandry systems where close and repeated human contact may create the likelihood of spillback exposure.

Initial work in Malaysia focused almost exclusively on human infections, and surveillance of spillback into animals lagged behind [17]. To address this gap, previous sero‐surveillance demonstrated substantial exposure to SARS‐CoV‐2 in companion animals, with neutralizing antibodies detected in approximately one quarter of dogs and cats using a species‐independent surrogate virus neutralization test (sVNT) [8]. A subsequent extension of the study to free‐ranging wild rodents provided the first serological evidence of SARS‐CoV‐2 spillback into *Sundamys muelleri* and *Rattus rattus* in Sarawak, although there was no indication of sustained rodent‐to‐rodent transmission [18]. Together, these findings confirm that reverse zoonotic transmission from humans to animals has occurred in this region across both domestic and wildlife interfaces.

Building on this body of evidence, livestock represent a logical yet understudied extension of SARS‐CoV‐2 spillback investigations in Sarawak. In contrast to companion animals and free‐ranging rodents, livestock in this setting are commonly managed within smallholder or semi‐intensive systems, where close and repeated contact with farmers, as well as occasional interactions with companion animals and synanthropic rodents, are commonplace. Such interfaces create plausible opportunities for human‐to‐livestock exposure, particularly during periods of intense community transmission. However, unlike pets and wildlife, the extent and epidemiological significance of SARS‐CoV‐2 exposure in livestock in Sarawak remain largely uncharacterized. To address this gap, the present study aimed to assess the seroprevalence of SARS‐CoV‐2 neutralizing antibodies in key livestock species and to provide baseline animal serological data that may inform future integrated One Health surveillance involving humans, animals, and environments.

## 2. Materials and Methods

A total of 180 archived plasma samples from five livestock species collected across multiple locations in Sarawak between June and September 2024 were obtained from the Sarawak Veterinary Diagnostic Service Laboratory. The sampled species comprised pigs (*Sus scrofa domesticus*, *n* = 39), buffalo (*Bubalus bubalis*, *n* = 36), sheep (*Ovis aries*, *n* = 35), goats (*Capra hircus*, *n* = 35), and cattle (*Bos taurus*, *n* = 35). Animals were categorized by species, age group (juvenile or adult), and sex at the time of sampling (Table 1). All plasma samples had been submitted routinely for standard veterinary diagnostic testing; no additional animal handling or sampling was performed for this study. Samples were anonymized, aliquoted as required, and stored at −20°C until analysis.

**Table 1:** Demographic characteristics of livestock collected in Sarawak that was included in this study.

Variable	Category	Positivity/total sample (%; 95%CI)
Species	Pigs	1/39 (2.6; 0.1–13.5)
	Buffalo	3/36 (8.3; 1.8–22.5)
	Sheep	10/35 (28.6; 14.6–46.3)
	Goat	17/35 (48.6; 31.4–66.0)
	Cattle	4/35 (11.4; 3.2–26.7)

Location	Serian	4/46 (8.7)
	Limbang	3/36 (8.3)
	Kota Samarahan	5/31 (16.1)
	Sri Aman	8/29 (27.6)
	Bintulu	9/19 (47.6)
	Sibu	4/7 (57.1)
	Sarikei	0/6 (0.0)
	Betong	2/6 (33.3)

Age group	Adult	26/141 (18.4)
	Juvenile	9/39 (23.1)

Sex	Female	27/144 (18.8)
	Male	8/36 (22.2)
	Total	35/180 (19.4; 13.9–26.0)

Neutralizing antibodies against SARS‐CoV‐2 were detected using the cPASS SARS‐CoV‐2 Neutralization Antibody Detection Kit (GenScript), a sVNT that measures the ability of antibodies to block the interaction between the viral spike receptor‐binding domain (RBD) and the human angiotensin‐converting enzyme 2 (ACE2) receptor [8, 18, 19]. The assay was previously used to screen for neutralizing SARS‐CoV‐2 antibodies in cattle, sheep, goat, and pig (and wild boar), and bison (family *Bovidae*, same family as buffalo) [16, 20–23].

The assay was performed according to the manufacturer’s instructions. Briefly, serum samples were incubated with horseradish peroxidase (HRP)‐conjugated RBD and then added to microplates precoated with human ACE2. After the incubation and washing steps, bound HRP‐RBD was detected using a chromogenic substrate, and absorbance was measured spectrophotometrically. Results were expressed as percentage inhibition, calculated relative to the negative controls. Samples with inhibition values ≥30% were interpreted as positive for SARS‐CoV‐2 neutralizing antibodies.

Descriptive statistics were used to summarize animal demographics and serological findings and are presented as frequencies and percentages. Data was compiled and curated in Microsoft Excel prior to analysis. Seroprevalence was calculated as the proportion of seropositive animals within each subgroup, and data were presented in frequencies and percentages.

During preliminary analyses, sparse data, and quasi‐complete were observed, especially within variables in the species and location categories. Due to these conditions, conventional maximum likelihood estimation would likely be biased, leading to unreliable odds ratios and confidence intervals (CIs). To address this issue, animal species were grouped according to their dietary profile, with pigs classified as small omnivores, goats and sheep as small ruminants, and cattle and buffalo as large ruminants. Sampling locations, on the other hand, were clustered according to their geographical location in Sarawak, with Kota Samarahan and Serian classified as Western Sarawak, Sri Aman and Betong as Central Sarawak, and Sibu, Sarikei, Bintulu, and Limbang as Northern Sarawak.

Additionally, to identify the factors independently associated with SARS‐CoV‐2 seropositivity, univariate and Firth’s multivariate penalized logistic regression were applied. The results were reported as crude odds ratios (cOR) and adjusted odds ratios (aOR) with the 95% CIs. The Firth’s penalized logistic regression was applied for the multivariable analysis in this study, as it uses a penalized likelihood approach prior to reduce small‐sample bias and provides finite, stable estimates even in the presence of separation. All statistical analyses were conducted in R Studio v2024.12 using the “logistf” package, and figures were generated using matplotlib v3.7.5. A two‐sided *p*‐value < 0.05 was considered statistically significant in this study.

## 3. Results

**Figure 1:**
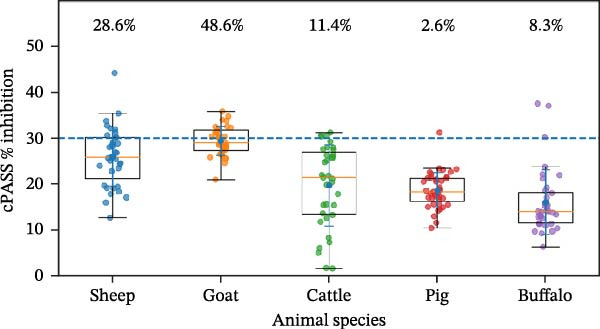
cPASS inhibition results by animal species. Distribution of SARS‐CoV‐2 surrogate virus neutralization assay (cPASS) inhibition values among different animal species. Box plots represent the median and interquartile range (IQR), with whiskers indicating data spread. Jittered points represent individual serum samples. Blue markers denote mean inhibition values with error bars indicating ± standard deviation (SD). The dashed horizontal line indicates the assay cut‐off value for seropositivity (30% inhibition). Seroprevalence indicated in percentage (%) above the respective box plots.

A total of 180 livestock were included in the study. A higher proportion of samples were collected from female livestock (80%), adults (78.33%), and from livestock in Serian (25.56%). Overall, 35/180 animals were seropositive, giving a seroprevalence of 19.4%. When stratified by species, seroprevalence was highest in goats (48.6%, 17/35), followed by sheep (28.6%, 10/35), cattle (11.4%, 4/35), buffalo (8.3%, 3/36), and pigs (2.6%, 1/39) (Table 1). Notably, the positive samples were predominantly low‐level positives, with inhibition values only modestly exceeding the positivity threshold (Figure 1).

**Figure 2:**
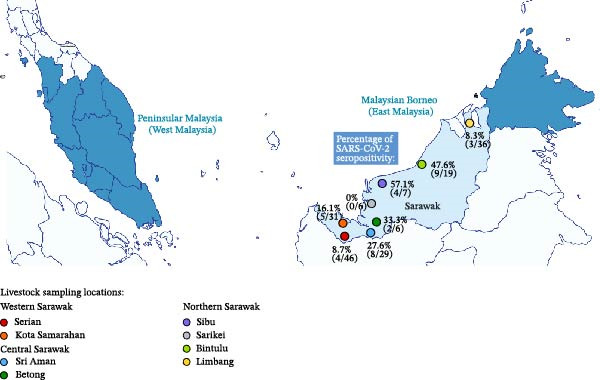
The sampling locations in Malaysia and the percentage of SARS‐CoV‐2 seropositivity in the studied animals.

Univariate analysis identified animal type and location as potential factors associated with seropositivity towards SARS‐CoV‐2 (Table 2). Small ruminants had significantly higher odds of seropositivity compared to large ruminants and small omnivores (cOR 5.74, CI 2.30–14.36, *p* < 0.001). Whereas, animals sampled from Western Sarawak had significantly lower odds of seropositivity compared to Central Sarawak and Northern Sarawak (cOR 0.33, CI 0.12–0.91, *p* < 0.03) (Figure 2). No significant associations were observed for age and sex.

**Table 2:** Univariate and multivariate analyses of risk factors associated with the seroprevalence of SARS‐CoV‐2 amongst farm animals.

Variable	Category	df	cOR (95% CI)	*p*‐Value	aOR (95% CI)	*p*‐Value
Animal diet	Large ruminant	2	Reference		Reference	
	Small ruminant	—	5.74 (2.30–14.36)	<0.001 ^∗^	10.01 (3.03–33.06)	<0.001 ^∗^
	Small omnivore	—	0.24 (0.03–2.03)	0.19	0.36 (0.03–2.049)	0.32
Location	Central Sarawak	2	Reference		Reference	
	Western Sarawak	—	0.33 (0.12–0.91)	0.03 ^∗^	3.14 (0.85–12.28)	0.08
	Northern Sarawak	—	0.77 (0.31–1.94)	0.58	2.87 (0.73–11.72)	0.13
Age group	Juvenile	1	Reference		Reference	
	Adult	—	0.75 (0.32–1.78)	0.52	1.19 (0.31–4.73)	0.80
Sex	Male	1	Reference		Reference	
	Female	—	0.801 (0.33–1.97)	0.64	1.58 (0.54–4.64)	0.44

*Note:* Reference categories: large ruminants (cattle and buffalo), Central Sarawak (Sri Aman and Betong), juvenile and male.

Abbreviations: 95% CI, 95% confidence interval; aOR, adjusted odds ratio; cOR, crude odds ratio; df, degree of freedom.

^∗^Denotes significance (*p*‐value < 0.05).

These variables were then subsequently included in Firth’s multivariate penalized logistic analysis regression model. After adjustment for location, age, and sex, animal diet remained independently associated with seropositivity. Small ruminants had significantly higher odds of seropositivity compared to large ruminants and small omnivores (aOR 10.01, CI 3.03–33.07, *p* < 0.001). The association between location and seropositivity was found to be no longer statistically significant in the adjusted model. Age and sex were not independently associated with seropositivity.

## 4. Discussion

This study provides evidence of prior SARS‐CoV‐2 exposure in livestock from Sarawak (Malaysian Borneo), with an overall seroprevalence of 19.4% (35/180). Seropositivity was strongly species‐dependent and geographically heterogeneous, while sex and age groups were not significantly associated with serostatus. Together, these findings are most consistent with sporadic spillback from humans into livestock rather than sustained livestock‐to‐livestock transmission.

A key feature of this dataset is that seropositive samples were predominantly low‐level positives. This pattern argues against widespread or recent infection in the sampled livestock and instead supports the interpretation of sporadic spillback exposure with weak and/or transient neutralizing antibody responses. Our findings are consistent with prior livestock sero‐surveillance using sVNTs. In a study involving 691 cattle, 698 sheep, and 707 goats in the United States, low sVNT seropositivity (1%–1.5%) was reported; however, sera exhibiting low‐level sVNT reactivity showed no detectable neutralizing activity when assessed using a conventional VNT. Thus, these findings underscore that low‐level sVNT signals may not necessarily correspond to robust functional neutralization in livestock and warrant cautious interpretation [16].

In addition, the detection of low seropositivity in this study aligns with the broader literature, which suggests that conventional livestock are generally low in susceptibility to SARS‐CoV‐2 infection, while occasional exposure and seroconversion may occur in real‐world settings [15]. Experimental infection data provide an important context. For example, an experimental study found cattle exhibit low susceptibility under experimental conditions with no evidence of intraspecies transmission to in‐contact animals, supporting that cattle are unlikely to contribute meaningfully to SARS‐CoV‐2 maintenance [24]. Likewise, the overall evidence base for pigs indicates minimal susceptibility to infection and limited or absent transmission in most studies, although low levels of virological or serological signals have been reported under specific experimental conditions [25–27].

Evidence of natural infection in small ruminants has historically been limited, with several studies reporting no antibodies detected even where contact with humans was plausible. For example, a study reported that no serological evidence of SARS‐CoV‐2 infection was found in sheep when comparing pre‐pandemic and pandemic‐era sera, suggesting that natural infection in sheep is uncommon [28]. More recently, the first serological evidence of natural infection in sheep and goats highlighted that the immune response may be weak and variable [29]. These studies collectively support two key points that align with our interpretation: (i) infection in sheep/goats can occur under natural conditions, but (ii) responses may be low‐titer and not consistently detected by stringent neutralization assays.

In this context, the markedly tenfold higher odds of seropositivity observed in small ruminants (sheep and goats) compared to large ruminants (cattle and buffalo) and small omnivores (pigs) in Sarawak may reflect that the potential of exposure exists at the human‐livestock interface rather than intrinsic susceptibility. Contextual drivers could include close daily handling, peri‐domestic husbandry, smallholder management, and the timing of sampling relative to community transmission waves. Importantly, the univariable association with location supports the possibility of clustered exposures, which is compatible with a spillback pattern.

The markedly low seropositivity in pigs in our study is consistent with experimental evidence, indicating that pigs are, at most, weakly susceptible. Although limited viral RNA detection and successful viral isolation from only one pig have been reported experimentally, inefficient infection may not result in sustained transmission [27]. This is further supported by studies that similarly demonstrate low susceptibility or lack of productive infection in pigs [25, 26]. Collectively, these findings support the interpretation that pigs are unlikely to act as reservoirs for SARS‐CoV‐2 and that field seropositivity, when present, is expected to be rare.

The low seroprevalence in cattle is compatible with experimental evidence of low susceptibility and absent in‐contact transmission [24]. Reports of SARS‐CoV‐2 seropositivity in buffalo are comparatively sparse, possibly reflecting the low susceptibility of this species, which may have resulted in limited surveillance prioritization. The low seroprevalence observed suggests that large ruminants, such as buffalo and cattle, are unlikely to be efficient hosts for sustained transmission. In practice, both cattle and buffalo may experience episodic exposure in high‐contact settings without becoming epidemiologically important amplifiers.

Nevertheless, the predominance of low‐level positives in our dataset necessitates a conservative interpretation. As noted above, sVNT‐positive livestock sera have not always shown neutralization in classical VNT formats in at least one large livestock study, highlighting potential differences in assay sensitivity, the biological magnitude of neutralizing responses in livestock, and timing relative to exposure [16]. In addition, while RBD‐based approaches are generally expected to be more specific than assays targeting more conserved antigens, background coronavirus exposure in livestock raises a reasonable concern about serological interference [30].

Although livestock are not considered major maintenance hosts for SARS‐CoV‐2, documenting exposure signals in livestock remains relevant for future One Health surveillance frameworks. Although this study did not include human and environmental testing, livestock serology may help identify husbandry systems where close and sustained human–animal contact may heighten exposure. This baseline data may be useful for designing future One Health studies that integrate livestock sampling with human infection history, farm management practices, and environmental surveillance. Second, even sporadic spillback events warrant attention because repeated cross‐species exposure creates opportunities for viral adaptation, even in species that are not considered major maintenance hosts [16]. The marked species gradient in our study suggests that surveillance yield may be higher in small ruminants than in pigs, consistent with the experimental literature, indicating minimal permissiveness in pigs but variable susceptibility in sheep and goats.

This study is limited by its cross‐sectional design, which cannot establish the timing of exposure or directionality of transmission between humans, animals, or the farm environment. The absence of parallel molecular testing, such as RT‐PCR, precludes the assessment of active infection and viral shedding at the time of sampling. Hence, the findings of this study should be interpreted as evidence of serological exposure rather than confirmation of the current infection. In addition, the clustering of positive results near the assay cut‐off highlights the need for confirmatory testing and careful interpretation, given that weak sVNT reactivity in livestock may not always be corroborated by VNT. Farm‐level clustering and detailed exposure covariates (e.g., household COVID‐19 history, animal movement, and husbandry intensity) were not incorporated and could explain spatial heterogeneity and species‐specific patterns in future work.

## 5. Conclusion

This study identified predominantly low‐level seropositivity against SARS‐CoV‐2 among livestock in Sarawak, Malaysia, consistent with sporadic spillback events from humans rather than widespread or sustained transmission within livestock populations. These findings are concordant with accumulating experimental and field evidence indicating that common livestock species are generally not competent hosts for SARS‐CoV‐2. Nevertheless, given the dynamic evolution of SARS‐CoV‐2 and the repeated opportunities for cross‐species exposure at the human–animal interface, periodic, risk‐based surveillance of livestock remains warranted to detect potential changes in host susceptibility or transmission patterns over time.

## Author Contributions

Cheng Siang Tan conceived the idea, planned the experiment, analyzed the data, and drafted the manuscript. Vaenessa Noni and Ahmad Syatir Tahar carried out the experiments and curated the data. Carolynna Buyau James and George Bobby coordinated sample inventory and planned the experiment. Jackie Peter coordinated sample inventory.

## Funding

This research received no specific grant from any funding agency in the public, commercial, or not‐for‐profit sectors.

## Disclosure

All authors reviewed and approved the manuscript.

## Ethics Statement

The study and method were approved by the Universiti Malaysia Sarawak Animal Ethics Committee (UNIMAS/AEC/2025/17).

## Conflicts of Interest

The authors declare no conflicts of interest.

## Data Availability

The data that support the findings of this study are available from the corresponding author upon reasonable request.
